# THE SUPPORT OF AUTONOMY FOR NURSING HOME RESIDENTS WITH DEMENTIA: OBSERVATION OF MORNING CARE

**DOI:** 10.1093/geroni/igz038.285

**Published:** 2019-11-08

**Authors:** Linda J Hoek, Hilde Verbeek, Erica De Vries, Jolanda C Van Haastregt, Ramona Backhaus, Jan P Hamers

**Affiliations:** 1 Department of Health Services Research, Care and Public Health Research Institute (CAPHRI), Faculty of Health, Medicine and Life Sciences,, Maastricht, Netherlands; 2 Department of Health Service Research, Care and Public Health Research Institute (CAPHRI), Maastricht University, Netherlands, Maastricht, Limburg, Netherlands

## Abstract

People with dementia in nursing homes need their social environment in supporting their autonomy. This study explored how this relational autonomy is supported by staff for residents with dementia during morning care in nursing homes. Structured observations (n=1815) were carried out to assess how resident choice is supported within staff-resident interaction. Observation of morning care consisted of four main categories: ‘getting up’, ‘physical care’, ‘physical appearance’ and ‘breakfast’. In addition, qualitative field notes were taken to support observations. In total, 55 residents with dementia were included from eight nursing home wards in The Netherlands. Results indicated that resident autonomy during morning care was only limitedly supported. Individual staff members took over tasks, regardless of resident’s individual capabilities to make a choice. Staff controlled resident’s choice for almost all observed categories. The findings of this study implicate that person-centered care during morning routine can be improved by addressing individual needs

